# Description of karyotype in *Hypostomus regani* (Ihering, 1905) (Teleostei, oricariidae) from the Piumhi river in Brazil with comments on karyotype variation found in *Hypostomus*

**DOI:** 10.3897/compcytogen.v5i2.964

**Published:** 2011-06-01

**Authors:** Ernani de Oliveira Mendes-Neto, Marcelo Ricardo Vicari, Roberto Ferreira Artoni, Orlando Moreira-Filho

**Affiliations:** 1 Universidade Federal de São Carlos. Depto. Genética e Evolução. São Carlos, SP, Brazil; 2 Universidade Estadual de Ponta Grossa. Depto. Biologia Estrutural, Molecular e Genética. Ponta Grossa, PR, Brazil

**Keywords:** cytotaxonomy, karyotype diversification, rDNA

## Abstract

The paper represents a comparative cytogenetic analysis of three populations of *Hypostomus regani* in Brazil.Two populations belong to the Upper Paraná River Basin and the third one, the karyotype of which is described for the first time, was probably introduced into the São Francisco River Basin through transposition from the Piumhi River. Karyotype features of populations of *Hypostomus regani* from the Piracicaba and Tietê River Basins are also discussed. The occurrence of *Hypostomus regani* in the São Francisco River Basin is reported for the first time here. The study also revealed distinct differences in the location of the Ag-NORs between the analyzed populations that enable individuals from the Piumhi River, Mogi-Guaçu River and Tietê River to be distinguished from one another. Thus, the data obtained indicate the possibility of geographic variation fixing different karyotypes even in the same basin of origin.

## Introduction

The family Loricariidae is the second most numerous among fish, with 716 species distributed among 96 genera (Ferraris 2007). These fish are endemic to the Neotropics, occurring from Costa Rica to Argentina (Reis et al. 2003). This considerable diversity has resulted in constant identification problems and new species have frequently been described (Pereira and Oyakawa 2003, Cardoso and Silva 2004). Recent studies have revealed that the taxonomy of Loricariidae remains poorly resolved (Armbruster 2004), but six subfamilies are recognized: Loricariinae, Hypoptopomatinae, Hypostominae, Neoplecostominae, Lithogeneinae and Delturinae (Reis et al. 2006). Although Reis et al. (2003) consider Loricariidae to be the largest family of catfish in the world, little is known regarding the constitution and organization of the karyotype in this group, which exhibits a tendency to show quite divergent karyotypes (Artoni and Bertollo 2001).

*Hypostomus* Lacépède, 1803 is the largest genus of the armored catfish family Loricariidae, with approximately 120 nominal species (Weber 2003). It is also one of the better characterized genera among the loricariids from the cytogenetic standpoint, revealing a variation in diploid number from 2n = 54 in *Hypostomus plecostomus* (Linnaeus, 1758) (Muramoto et al. 1968, cited in Artoni and Bertollo 2001) to 2n = 84 in *Hypostomus* sp. 2 (Cereali et al. 2008). Although some trends of the karyotype evolution have been described in *Hypostomus* (Artoni and Bertollo 2001, Kavalco et al. 2005, Alves et al. 2006, Kavalco et al. 2005, Milhomem et al. 2010), especially such features as increase in the diploid number by centric fission (Artoni and Bertollo 1996), the number of karyotyped species is still very small compared to the diversity of species known in this genus.

In the present study, a comparative cytogenetic analysis was carried out on three different populations of *Hypostomus regani* (Ihering, 1905). Two populations are from the Upper Paraná River Basin and the other, the karyotype of which is described for the first time, was probably introduced into the São Francisco River Basin through the transposition of the Piumhi River. The aim was to investigate the karyotype in these populations, seeking chromosomal characters potentially important for understanding the taxonomy and biogeography of the species.

## Material and methods

**Figure 1.**
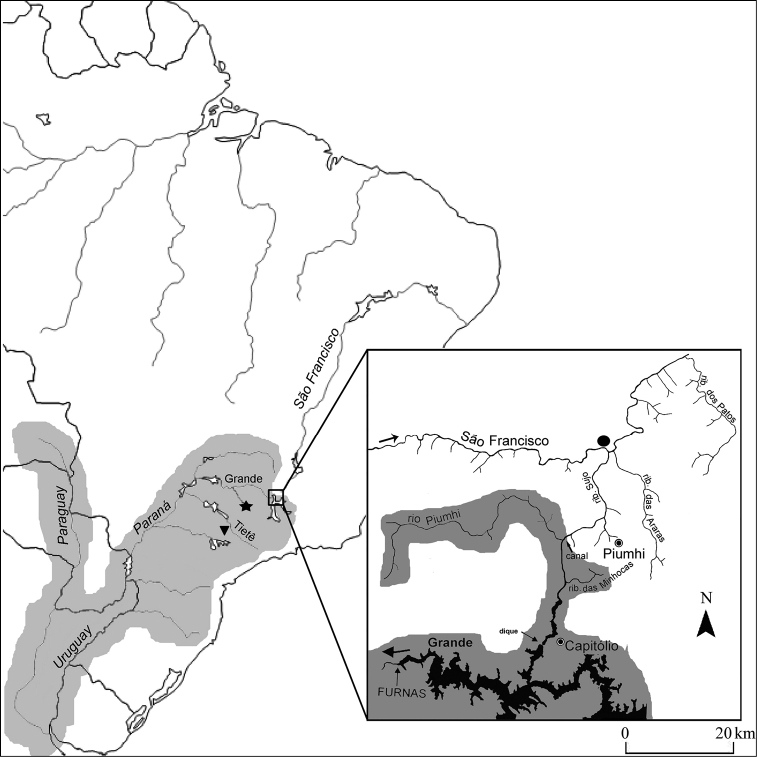
Map of Brazil highlighting the large hydrographic basin of the Paraná-Paraguai system, area of natural distribution of *Hypostomus regain*. Detail: area of the divider of the waters of the Upper Paraná and São Francisco River Basins, altered by the transposition of the Piumhi River (with original drainage to the Grande River in the Upper Paraná Basin) to the Upper São Francisco Basin through an artificial channel that links it to the Sujo River (tributary of the São Francisco River). Star (★) and triangle (▼) indicate sampling sites for the *Hypostomus regani* populations studied by (Artoni and Bertollo (1996, 2001) and Alves et al. (2006) in the Mogi-Guaçu and Tietê River Basins, respectively; circle (●) indicates the sampling site for the *Hypostomus regani* specimens analyzed in the present study in the confluence of the Rio Piumhi with to Rio São Francisco, upper Rio São Francisco basin.

Sixteen specimens of *Hypostomus regani* were examined (8 males and 8 females), collected from the mouth of the Piumhi River at the São Francisco River in the region of the municipality of Piumhi – MG, Brazil (20°20'31.0"S, 45°59'03.4"W, Alt.: 640 m), (Fig. 1, detail).According to C.H. Zawadzki (personal communication), *Hypostomus regani* is characterized as a species with a body covered by small, round, light-colored and generally well-defined spots. It has a high body and relatively long (narrow) head, large eyes and long dorsal fins, generally with rays reaching the spine of the adipose fin when adpressed. It has plates on the abdomen, except for very young specimens. Although distributed throughout the Paraná-Paraguay Basin, its type locality is the Piracicaba River in the state of São Paulo, Brazil.

The specimens were identified and deposited in the Museu Nacional do Rio de Janeiro (MNRJ 32778; MNRJ 32782; MNRJ 32787). The collection authorization (number 472897) was granted by IBAMA [Brazilian Environmental Protection Agency] and the fishing license (number 091/07) was granted by the Instituto Estadual de Floresta de Minas Gerais, Brazil.

Karyotype data on the *Hypostomus regani* populations studied by Artoni and Bertollo (1996), Artoni and Bertollo (2001) from the Mogi-Guaçu River Basin and by Alves et al. (2006) from the Tietê River Basin (Fig. 1) were also accessed (Table 1).

**Table 1.** Chromosomal data of the *Hypostomus regani* populations; from Artoni and Bertollo (2001) (Ref. 1), Alves *et al.* (2006) (Ref. 2) and new data from the population introduced into the São Francisco River Basin (Ref. 3).

Locality	2n/FN	Formula	Ag-NOR	Ref.
Rio Mogi-Guaçu, Rio Mogi-Guaçu basin	72/116	10m+20sm+42st/a	Multiple1 pair “a” large1 par “st” small	1
Rio Araquá, Rio Tiete basin	72/116	12m+18sm+26st+16a	Multiple2 pairs “a” largies	2
Confluence of the Rio Piumhi with to Rio São Francisco, upper Rio São Francisco basin	72/116	8m+16sm+20st+28a	Simple1 pair “st” large	3

Chromosome preparations were obtained from cells from the anterior portion of the kidney, using *in vivo* treatment with colchicine (Bertollo et al. 1978). Nucleolus organizer regions (NORs) were detected using silver nitrate (Ag-NORs), based on the method described by Howell and Black (1980). C-positive heterochromatin was analyzed using the C-banding method (Sumner 1972).

Fluorescent *in situ* hybridization (FISH) was employed to locate ribosomal genes in the chromosomes. An 18S rDNA probe from the fish *Prochilodus argenteus* (Agassiz, 1829)(Hatanaka and Galetti Jr. 2004) and a 5S rDNA probe from the fish *Leporinus elongatus* Valenciennes, 1849 (Martins and Galetti Jr. 1999) were used to map the rDNA sites on the chromosomes. Both probes were labeled with 14-dATP biotin by nick translation, following the manufacturer’s instructions (Bionick Labeling System – Invitrogen). Amplification and detection of the hybridization signals was carried out using the avidin-FITC and anti-avidin biotin complex system (Sigma). FISH signals were viewed based on the method described by Pinkel et al. (1986) and analyzed under an epifluorescence microscope (Olympus BX51). The images of the chromosomes were captured using the CoolSNAP-Pro software program (Media Cybernetics).

Approximately 30 metaphases from each specimen were analyzed in order to determine the modal diploid number (2n), fundamental number (FN) and karyotype formula. The chromosomes were identified based on the approach described by Levan et al. (1964) and classified in the karyotype as metacentric (m), submetacentric (sm), subtelocentric (st) and acrocentric (a).

## Results

**Figure 2.**
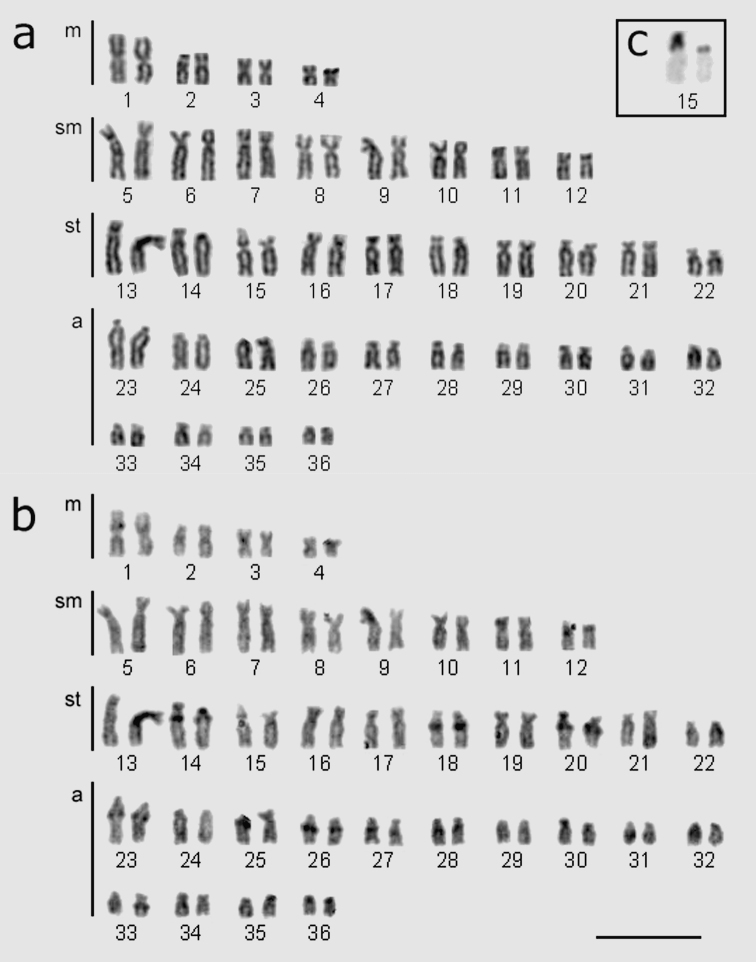
*Hypostomus regani* karyotypes from the confluence of the Piumhi and São Francisco Rivers **a** chromosome stained with Giemsa and **b** sequentially labeled by C-banding; **c** box indicates chromosome pair labeled with silver nitrate locating the nucleolus organizer regions (pair no. 15). Bar = 10 µm.

All the *Hypostomus regani* specimens analyzed in the present study had 2n = 72 chromosomes with a karyotype formula 8m+16sm+20st+28a. The number of arms was FN = 116 (Table 1, Fig. 2a).

Constitutive heterochromatin was distributed in small blocks (Fig. 2,b). The interstitial region of the long arms of subtelocentric chromosomes pairs 14, 18 and 20 as well as acrocentric pairs 23, 26, 27, 28 and 33 had quite evident blocks. Metacentric chromosome pair 1 had fainter labeling in the interstitial region of the short arm. Chromosome pairs 13, 15 and 36 had heterochromatic blocks in the centromeric region. Heterochromatin was located in the telomeric region of the long arm in chromosome pair 31. Nucleolar organizing regions (NORs) labeled by silver nitrate were only evident in the short arm of subtelocentric chromosome pair 15 (Fig. 2,c and 4,a). All cells analyzed exhibited heteromorphism in relation to the size of the Ag-NORs.

**Figure 3.**
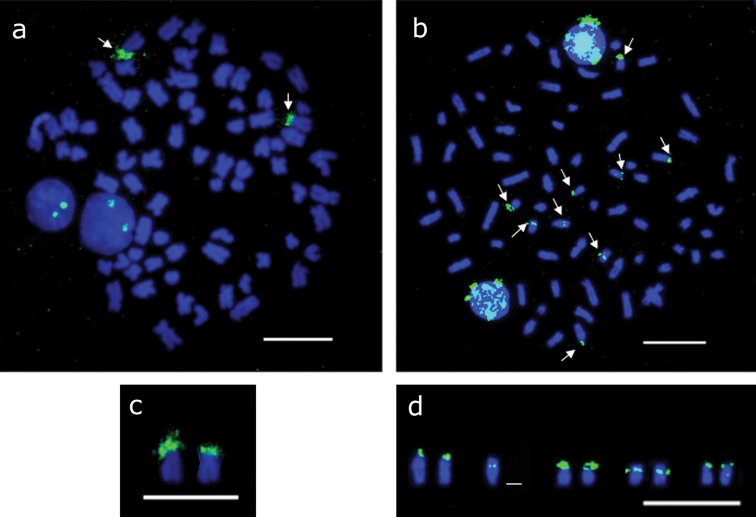
Mitotic metaphases in *Hypostomus regani* from the confluence of the Piumhi and São Francisco Rivers submitted to fluorescent *in situ* hybridization **a** showing two 18S rDNA sites (arrows) **b** nine 5S rDNA sites (arrows) **c** chromosomes bearing 18S rDNA sites **d** chromosomes bearing 5S rDNA sites. Bar = 10 µm.

Fluorescent *in situ* hybridization confirmed the presence of 18S rDNA coinciding with the Ag-NORs as well as the size heteromorphism of the sites (Figs 3,a-c). The 5s rDNA sites were located in four chromosome pairs: in the terminal region of the short arm of two acrocentric pairs; in the centromeric region of one submetacentric pair; and on another chromosome with no evident homologous labeling (Figs 3,b-d).

**Figure 4.**
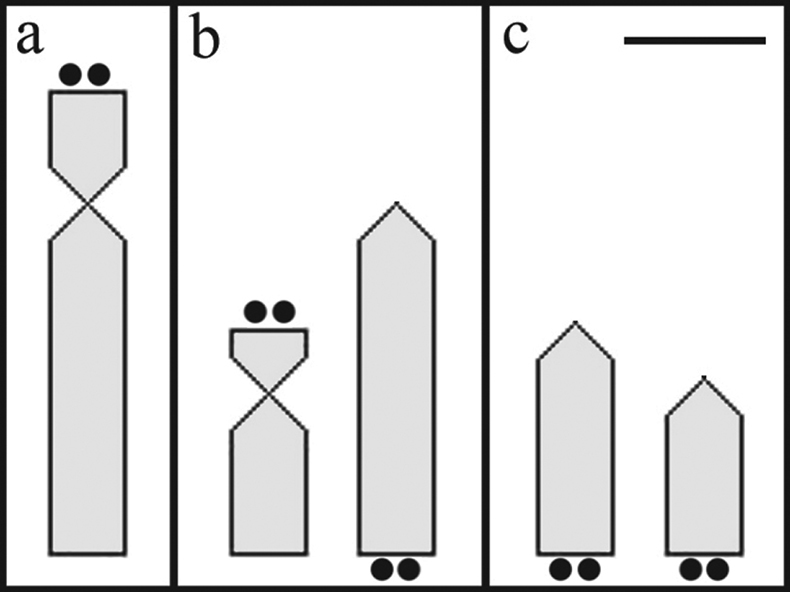
Idiogram of chromosomes bearing nucleolus organizer regions labeled by silver nitrate, comparing *Hypostomus regani* populations from the Piumhi River in the Upper São Francisco Basin **a** and Mogi Guaçu **b** and Tietê **c** Rivers in the Upper Paraná Basin. Bar = 1 µm.

## Discussion

The Piracicaba River, which is a tributary of the Upper Paraná River, is the type locality of *Hypostomus regani*, although the natural distribution of the species is related to the Paraná, Paraguay and Uruguay River Basins (Carvalho and Bockmann 2007). The occurrence of *Hypostomus regani* in the São Francisco River Basin is reported for the first time here and is added to the data from the available taxonomic sources (Isbrücker 1980, Montoya-Burgos 2003, Weber 2003, Armbruster 2004, Ferraris 2007). *Hypostomus regani* likely invaded the São Francisco River Basin after the transposition of the Piumhi River (Moreira-Filho and Buckup 2005). Although the field and taxonomic data confirm the origin of this species in the Upper Paraná River Basin, the data presented here indicate a divergence in the karyotype macrostructure that involves the possibility of geographic variation. The different cytotypes may have been isolated in allopatry approximately six million years ago (this dating is based on the origin time of the basin of San Francisco river) (Montoya-Burgos 2003). On the other hand, we must also consider the possibility of karyotype diversification occurring within the same hydrographic basin, like the Upper Paraná River Basin.

Karyotype differences in natural fish populations that inhabit the same hydrographic basin have been found, for instance, in the genus *Astyanax* Baird et Girard, 1854. E.g. there are at least three different cytotypes of *Astyanax* prope *fasciatus* (Cuvier, 1819) living in sympatry in the Upper Tibagi River, which is a tributary of the Paraná River (Artoni et al. 2006). According to Artoni et al. (2009), events of geographic vicariance stemming from the history of the South American continent are among the factors to be considered in the karyotype diversification of Neotropical freshwater fish. The authors also consider the evolutionary time for the fixation of chromosome rearrangements and the biology of species. We must stress here that *Hypostomus regani* is not a great migrator, as with the majority of Loricariidae, the anatomy of which imposes difficulties on the movement of these fish in overcoming physical barriers, such as waterfalls (Paiva et al. 2005). This imposition favors the formation of more restricted population demes, which may accelerate the formation of new cytotypes within geographically isolated areas through the action of genetic drift and the restriction of gene flow. In *Ancistrus* Kner, 1854, for example, the great karyotypic variability may be related to biological and behavioral characteristics of these armored catfish that include microhabitat preferences, territoriality and specialized reproductive tactics, with consequences for the fixation of chromosomal rearrangements and speciation (Oliveira et al. 2009).

In a previous study, Artoni and Bertollo (2001) point to evolutionary trends for the karyotype of the subfamilies of Loricariidae. Among those that exhibit extensive variation in the diploid number, the genus *Hypostomus* stands out, with inter-species variation ranging from 2n = 54 to 84 chromosomes, which demonstrates the strong action of events of centric fission in the karyotype diversification of this group when compared to more basal forms found in sister groups of Hypostominae, such as *Liposarcus* Günther, 1864 (2n = 52), *Rhinelepsis* Agassiz, 1829 (2n = 54) and *Pogonopoma* Regan, 1904 (2n=54) (Artoni et al. 1999, Artoni and Bertollo 2001). The data present here for *Hypostomus regani*, in relation for the most basal *Hypostomus* species, also support the Artoni and Bertollo’s hypothesis (*op. cit.*) regarding the location and distribution of heterochromatin, especially in relation to the accumulation of equilocal and interstitial heterochromatic blocks preferentially located in subtelocentric and acrocentric chromosomes. Artoni and Bertollo (1999) propose that this tendency increases among species of *Hypostomus* that have higher diploid numbers as a consequence of likely translocations between non-homologous chromosomes in the interphase nucleus.

Especially regarding the species *Hypostomus regani*, we can highlight the location of nucleolus organizer regions (NORs) as a variable inter-population character. The results reveal distinct patterns of chromosome types and location that enable the *Hypostomus regani* population in the Piumhi River of the São Francisco Basin to be distinguished from the populations in the Mogi-Guaçu and Tietê Rivers analyzed by (Artoni and Bertollo (1996, 2001) and Alves et al. (2006), respectively. With these data on the gene activity of the NORs reinforced by the *in situ* chromosome location of the 18s rDNA sites (FISH), we may suggest that the colonizing *Hypostomus regani* individuals that invaded the São Francisco River Basin facilitated by the transposition of the Piumhi River (Moreira-Filho and Buckup 2005) may have originated from a population of this species that inhabits the Grande River Basin. In its turn, the last population is karyotypically distinct from other populations of this species that occur in the Upper Paraná River Basin (Fig. 4).

The results obtained with the chromosomal location of 5S gene do not currently allow any evolutionary inferences. However this cytotaxonomical marker may be important in future studies, especially regarding the number and location of this class of ribosomal DNA in *Hypostomus*.

Besides the identification of a population of *Hypostomus regani* belonging to the native ichthyofauna of the Piumhi River basin, originally founded from the ichthyofauna of the Upper Paraná River, we also verified the introduction of an exotic species in the São Francisco River Basin, with unpredictable consequences for the homeostasis of this environment.
